# Iron oxide magnetic nanoparticles highlight early involvement of the choroid plexus in central nervous system inflammation

**DOI:** 10.1042/AN20120081

**Published:** 2013-03-28

**Authors:** Jason M. Millward, Jörg Schnorr, Matthias Taupitz, Susanne Wagner, Jens T. Wuerfel, Carmen Infante-Duarte

**Affiliations:** *Institute for Medical Immunology, Charité-Universtitätsmedizin Berlin, Berlin, Germany; †Experimental and Clinical Research Center (a joint cooperation between the Charité Medical Faculty and the Max-Delbrück Center for Molecular Medicine), Berlin, Germany; ‡Department of Radiology, Charité-Universitätsmedizin Berlin, Berlin, Germany; §Cluster of Excellence NeuroCure, Charité-Universitätsmedizin Berlin, Berlin, Germany; ∥Institute of Neuroradiology, University Medicine Göttingen, Göttingen, Germany

**Keywords:** choroid plexus, experimental autoimmune encephalomyelitis, iron oxide nanoparticles, magnetic resonance imaging, multiple sclerosis, BBB, blood–brain barrier, CNS, central nervous system, CSF, cerebrospinal fluid, EAE, experimental autoimmune encephalomyelitis, FOV, field of view, GAG, glycosaminoglycan, Gd-DTPA, gadopentetate dimeglumine, GFAP, glial fibrillary acidic protein, H&E, haematoxylin and eosin, MRI, magnetic resonance imaging, PLP, proteolipid peptide, VSOP, very small superparamagnetic iron oxide nanoparticles

## Abstract

Neuroinflammation during multiple sclerosis involves immune cell infiltration and disruption of the BBB (blood–brain barrier). Both processes can be visualized by MRI (magnetic resonance imaging), in multiple sclerosis patients and in the animal model EAE (experimental autoimmune encephalomyelitis). We previously showed that VSOPs (very small superparamagnetic iron oxide particles) reveal CNS (central nervous system) lesions in EAE which are not detectable by conventional contrast agents in MRI. We hypothesized that VSOP may help detect early, subtle inflammatory events that would otherwise remain imperceptible. To investigate the capacity of VSOP to reveal early events in CNS inflammation, we induced EAE in SJL mice using encephalitogenic T-cells, and administered VSOP prior to onset of clinical symptoms. In parallel, we administered VSOP to mice at peak disease, and to unmanipulated controls. We examined the distribution of VSOP in the CNS by MRI and histology. Prior to disease onset, in asymptomatic mice, VSOP accumulated in the choroid plexus and in spinal cord meninges in the absence of overt inflammation. However, VSOP was undetectable in the CNS of non-immunized control mice. At peak disease, VSOP was broadly distributed; we observed particles in perivascular inflammatory lesions with apparently preserved glia limitans. Moreover, at peak disease, VSOP was prominent in the choroid plexus and was seen in elongated endothelial structures, co-localized with phagocytes, and diffusely disseminated in the parenchyma, suggesting multiple entry mechanisms of VSOP into the CNS. Thus, using VSOP we were able to discriminate between inflammatory events occurring in established EAE and, importantly, we identified CNS alterations that appear to precede immune cell infiltration and clinical onset.

## INTRODUCTION

Inflammatory diseases of the CNS (central nervous system), such as multiple sclerosis, are characterized by the infiltration of immune cells from the periphery. To gain entry to the CNS, immune cells must cross the BBB (blood–brain barrier), a complex multi-step process that involves interaction with numerous cellular and non-cellular elements (Owens et al., 2008). Disruption of the BBB associated with active inflammation can be visualized *in vivo* by contrast-enhanced MRI (magnetic resonance imaging). Gd-DTPA (gadopentetate dimeglumine)-enhancing lesions are also detectible in the mouse model of multiple sclerosis, EAE (experimental autoimmune encephalomyelitis), in which it was histologically confirmed that the MRI lesions corresponded to focal inflammation and BBB breakdown (Smorodchenko et al., 2007). Nevertheless, discrepancies remain between the presence of Gd-enhancing lesions and the clinical severity in both multiple sclerosis and EAE (Barkhof, 2002; Okuda et al., 2009). To overcome this so-called clinical–radiological paradox (Wuerfel et al., 2007) superparamagnetic iron oxide nanoparticles with strong magnetic susceptibility effects were applied (Weinstein et al., 2010). Unlike Gd-DTPA, iron oxide particles are efficiently taken up by phagocytic cells, and partition to the liver, spleen and lymph nodes. The capacity of iron oxide particles to be phagocytosed has enabled the use of MRI to track macrophage dynamics in EAE studies (Oude Engberink et al., 2010; Rausch et al., 2003, 2004). VSOPs (very small superparamagnetic iron oxide particles) are electrostatically stabilized with a citrate coating, and present with a hydrodynamic diameter of only 7 nm, which is much smaller compared with other iron oxide particles (Taupitz et al., 2000; Wagner et al., 2002). Originally developed as a blood pool contrast agent (Schnorr et al., 2004), we previously showed VSOP to be efficiently phagocytosed (Stroh et al., 2006) and to have the potential to detect lesions in EAE (Wuerfel et al., 2007). In addition to being phagocytosed, we previously showed that VSOP could efficiently extravasate and highlight both acute BBB disruption and persistent lesions, being detectable by MRI in the brain up to 20 days post application (Tysiak et al., 2009; Wuerfel et al., 2007). Thus VSOP may be regarded as having properties intermediate between Gd-based contrast agents and other larger-sized iron oxide nanoparticles.

In a previous study, we compared lesions characterized by VSOP with those enhancing Gd-DTPA in EAE, and showed that each Gd-DTPA-enhancing lesion correlated with T2*-signal loss after VSOP injection; however, numerous instances of lesions were detected only with VSOP and not with Gd-DTPA (Tysiak et al., 2009). We hypothesized that these lesions detected only with VSOP may represent inflammatory lesions at a relatively early stage, in which the immune cells are confined to the perivascular space. To further investigate our hypothesis, we here administered VSOP to EAE mice both prior to clinical disease onset, when only very early inflammatory events may occur, and at peak disease. We undertook a detailed histological examination of the spinal cord and the brain parenchyma to investigate the tissue distribution of VSOP at early and peak neuroinflammation.

## MATERIALS AND METHODS

### Induction of EAE

All procedures were performed in accordance with protocols approved by the local animal welfare committee (LAGeSo) in accordance with national and international guidelines to minimize discomfort to animals (86/609/EEC). Female SJL/J mice were purchased from Janvier and were housed under standard conditions. For induction of passive EAE, donor mice were immunized subcutaneously with 200 μg of PLP (proteolipid protein) peptide_139-151_ purity >95% (Pepceuticals Ltd) together with Complete Freund's adjuvant and heat-killed *Mycobacterium tuberculosis* (H37Ra, Difco). Ten days after immunization, mice were killed by cervical dislocation and the draining lymph nodes were collected. Lymph node cells were isolated by macerating the tissue through a 100 μm mesh. The cells were incubated at 37°C for 4 days in the presence of 12.5 μg/ml PLP peptide in RPMI 1640 medium [supplemented with 2 mM l-glutamine, 100 units/ml penicillin, 100 μg/ml streptomycin and 10% FBS (fetal bovine serum)]. Then 5×10^6^ lymphocyte blasts were injected intravenously into a total of 34 syngenic recipient mice. Mice were assigned a clinical score daily: 0, no disease; 1, tail weakness; 2, paraparesis; 3, paraplegia; 4, paraplegia with forelimb weakness; 5, moribund or dead animals.

### MRI procedures

MRI was done using a 7 Tesla Bruker Pharmascan 70/16 rodent MR scanner (Bruker Biospin), applying a 20 mm RF-Quadrature-Volume head coil. Mice were anaesthetized with 1.5–2.0% isofluorane in 30% O_2_ and 70% N_2_O administered via face mask, under continuous ventilation monitoring (Bio Trig System, Bruker Biospin). The animals were placed on a bed with circulating heated water to maintain constant body temperature at 37°C.

Sixteen mice underwent axial and coronal T1- and T2*-weighted native MRI to establish the pre-contrast baseline. Subsequently, these mice received 0.2 mmol/kg Gd-DTPA (Magnevist, Bayer-Schering AG) intravenously and were immediately scanned again for contrast-enhanced T1-weighted imaging. After this, the mice received intravenous 0.2 mmol/kg VSOP (VSOP-R1 preparation batch 080610, produced by Charité Institute of Radiology with the following physicochemical properties: 16% citric acid (weight citric acid:iron); r1=18.54; r2=47.15 at 0.94 T; hydrodynamic diameter 90% of particles 5.6–8.7 nm; crystal size [TEM (transmission electron microscopy)]: 4.8±0.4 nm; pH adjusted with sodium hydroxide and in a final galenic formulation with 60 g/l mannitol and 2 g/l *N*-methylglucamine). Synthesis and physicochemical properties are comparable with VSOP-C184 which has already been tested in human clinical trials up to phase II (Wagner et al., 2011). After VSOP application, mice were returned to the home cage and T2*-weighted sequences were acquired 24 h later.

T1- and T2*-weighted images were acquired with the following parameters: axial T1w images (RARE, TE 10.5 ms, TR 804.1 ms, 0.5 mm slice thickness, matrix 256, FOV (field of view) 2.85 cm, four averages, 30 slices, scan time 6 min 51 s); coronal T1w images (RARE, TE 10.6 ms, TR 938.1 ms, 0.5 mm slice thickness, matrix 256, FOV 2.85 cm, four averages, 25 slices, scan time 8 min 32 s); axial T2*w images (FLASH, TE 7.2 ms, TR 619.7 ms, flip angle 30°, 0.44 mm slice thickness, matrix 256, FOV 2.85 cm, four averages, 40 slices, scan time 10 min 34 s); coronal T2*w images (FLASH, TE 7.2 ms, TR 386.2 ms, flip angle 30°, 0.43 mm slice thickness, matrix 256, FOV 2.85 cm, four averages, 25 slices, scan time 6 min 35 s). Data acquisition was done with Paravision 4.0 (Bruker Biospin).

### Histology

In all cases, mice were processed for histology 24 h after intravenous administration of VSOP. As a control, 10 unmanipulated SJL mice also received VSOP and were processed for histology the same way. After terminal anaesthesia, mice were transcardially perfused with 20 ml PBS, then with 20 ml zinc fixation solution (0.5% zinc acetate, 0.5% zinc chloride, 0.05% calcium acetate) (Schellenberger et al., 2011). Brains and spinal cords were then extracted and subsequently post fixed in zinc solution for 3 days at room temperature (25°C). The tissues were then cryoprotected by incubation overnight at 4°C in 30% sucrose in PBS, then embedded in O.C.T. and frozen in methylbutane with dry ice. The tissues were cut into 12 μm sections on a cryostat, and stained with H&E (haematoxylin and eosin) according to standard procedures.

For immunostaining, tissue sections were blocked with avidin, biotin and normal rabbit serum, then incubated overnight at 4°C with rabbit anti-GFAP (glial fibrillary acidic protein; Dako), rabbit anti-iba-1 (Wako) or rabbit anti-laminin (Novus) antibodies; all primary antibodies diluted 1:1000. The sections were then incubated with biotinylated goat anti-rabbit IgG antibody (Vector Laboratories), then streptavidin-conjugated peroxidase and visualized with Vector NovaRED Peroxidase substrate (Vector Laboratories). Prussian Blue staining for iron detection was done using Perl's method (incubation with 1% potassium hexacyanoferrate and 1% HCl), followed by counterstaining with Nuclear Fast Red.

## RESULTS

### VSOP distribution in CNS prior to EAE onset

**Figure 1:**
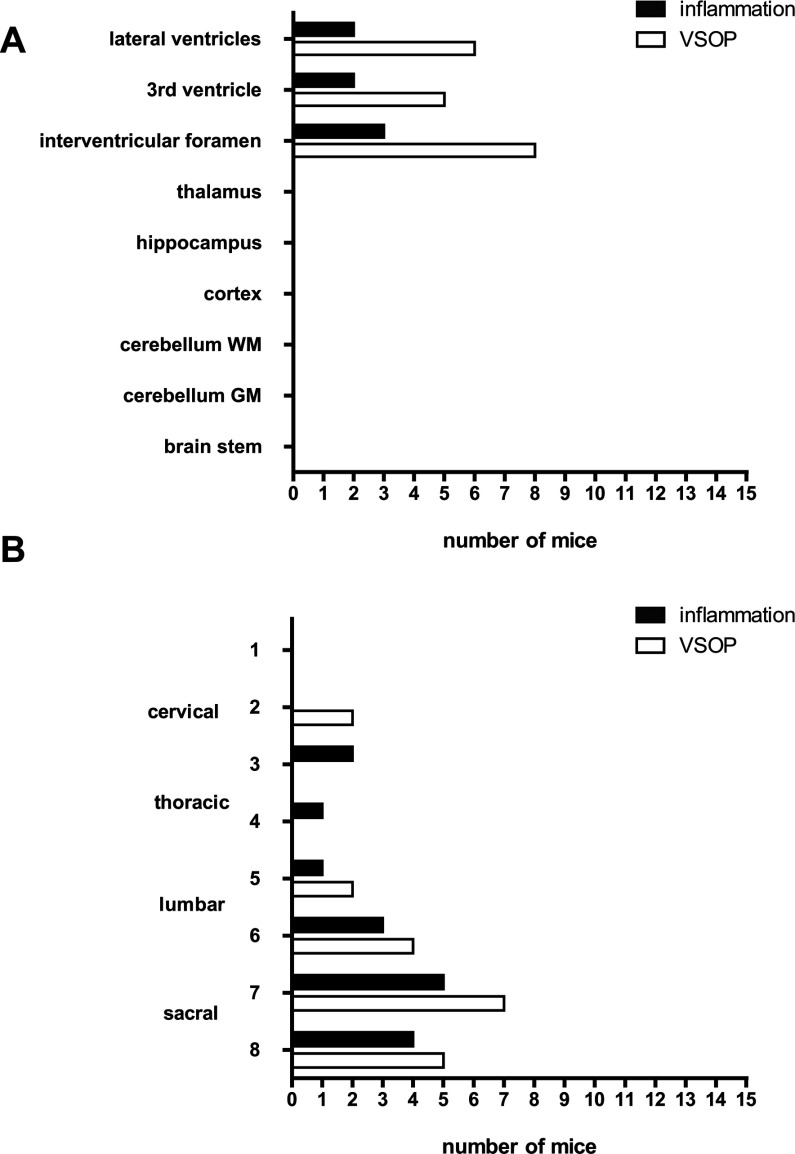
Histological detection of inflammation and VSOP prior to EAE clinical onset (4–6 days post transfer) The number of mice showing inflammation, defined as pathological accumulations of immune cells as revealed by H&E staining, and VSOP as shown by Prussian Blue staining, is indicated for various locations in the brain (**A**). The spinal cord (**B**) was cut into eight transverse segments spanning from the cervical to sacral zones. *n*=15.

In the present study, we aimed to evaluate the utility of VSOP to detect early events in EAE pathogenesis, prior to the onset of clinical signs. In a previous study, we administered VSOP to mice with established Gd-DTPA-enhancing lesions, and demonstrated that VSOP revealed lesions that were not detectable by Gd-DTPA (Tysiak et al., 2009). In the present study, we examined the CNS of mice between 4 and 6 days after adoptive transfer of encephalitogenic T-cells from PLP_139-151_-immunized donor mice – a time point that precedes the development of clinical disability. Figure 1 shows the distribution of inflammatory pathology and histological detection of VSOP in brain and spinal cord (Figures 1A and 1B, respectively). VSOP was detected in the brain in eight out of 15 mice (53%) at this pre-onset phase. At this early phase, the VSOP was seen exclusively in the choroid plexus in the lateral and third ventricles, and in the interventricular foramen (Figure 1A).

**Figure 2:**
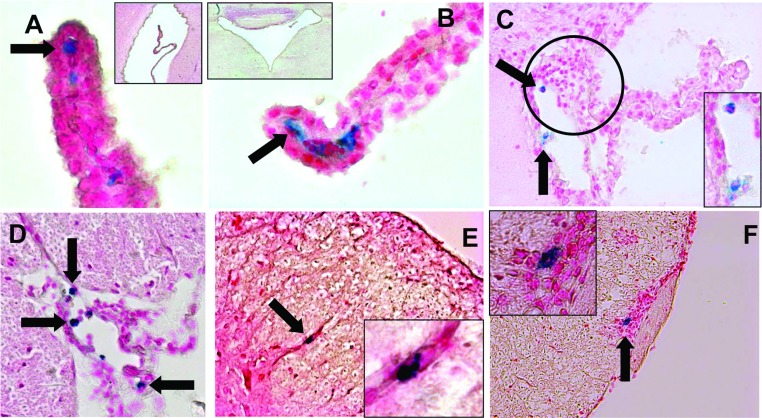
VSOP detected in CNS prior to EAE clinical onset (4–6 days post transfer) VSOP (blue staining) was observed in the stroma of the choroid plexus of the lateral (**A**) and fourth (**B**) ventricles (arrows), in the absence of inflammatory cells. VSOP was also seen in choroid plexus in the presence of mild inflammation (circle) (**C**). VSOP was detected in spinal cord meninges (**D**) and vessels (**E**) in the absence of inflammation, and in the presence of inflammatory cells (**F**). Original magnification: (**A**, **B**) ×400; (**C**–**E**) ×200; (**F**) ×100.

Of these eight mice presenting VSOP in the brain prior to EAE onset, five show VSOP in the absence of a pathological accumulation of immune cells as depicted in Figures 2(A) and 2(B). In these pictures, representative examples of the presence of VSOP, revealed by Prussian Blue iron staining located within the choroid plexus stroma (i.e. basal to the choroid epithelium), of the lateral ventricle (Figure 2A) and the fourth ventricle (Figure 2B) are shown. In these animals, the architecture of the choroid plexus appeared intact.

The three other mice showed signs of inflammation in the choroid plexus, defined as a pathological accumulation of immune cell infiltrates shown by H&E staining (indicated by the circle) and also partially disrupted tissue architecture as shown in Figure 2(C). No mice showed inflammation in any other brain regions at this pre-onset time point.

In the spinal cord (Figure 1B), VSOP was detected in seven out of 15 mice (46%), located in white matter and meninges. Mild inflammatory pathology could be seen in white matter and meninges in five out of 15 mice, predominantly in the lumbar and sacral regions, conforming to a pattern of ascending pathology (Figure 1B). Figure 2(D) shows a representative example of VSOP in the spinal cord meninges. As in the choroid plexus, VSOP was seen without a pathological accumulation of infiltrated immune cells in this area. Figure 2(E) shows an example of VSOP in a non-inflamed vessel in the spinal cord white matter. Figure 2(F) shows VSOP in the presence of a mild inflammatory lesion in the meninges.

As a control, we administered the same dose of VSOP to 10 unmanipulated SJL mice, and processed the tissues for histology in an identical manner. No VSOP was detectable in the spinal cord meninges, the choroid plexus or any other CNS structures in these mice, suggesting that the detection of VSOP in the pre-onset mice was related to early – otherwise not detectable – inflammatory processes. In healthy control mice, VSOP injected intravenously accumulate in the liver and are cleared primarily by its phagocytosing systems (Wagner et al., 2002). For all mice, liver tissue served as a positive control for detection of VSOP (not shown). Thus, in immunized animals and prior the appearance of clinical signs of EAE, VSOP accumulates in spinal cord as well as the choroid plexus and intraventricular foramen, also in the absence of apparent inflammation.

### VSOP-mediated visualization of brain inflammation at disease peak using MRI and histology

**Figure 3:**
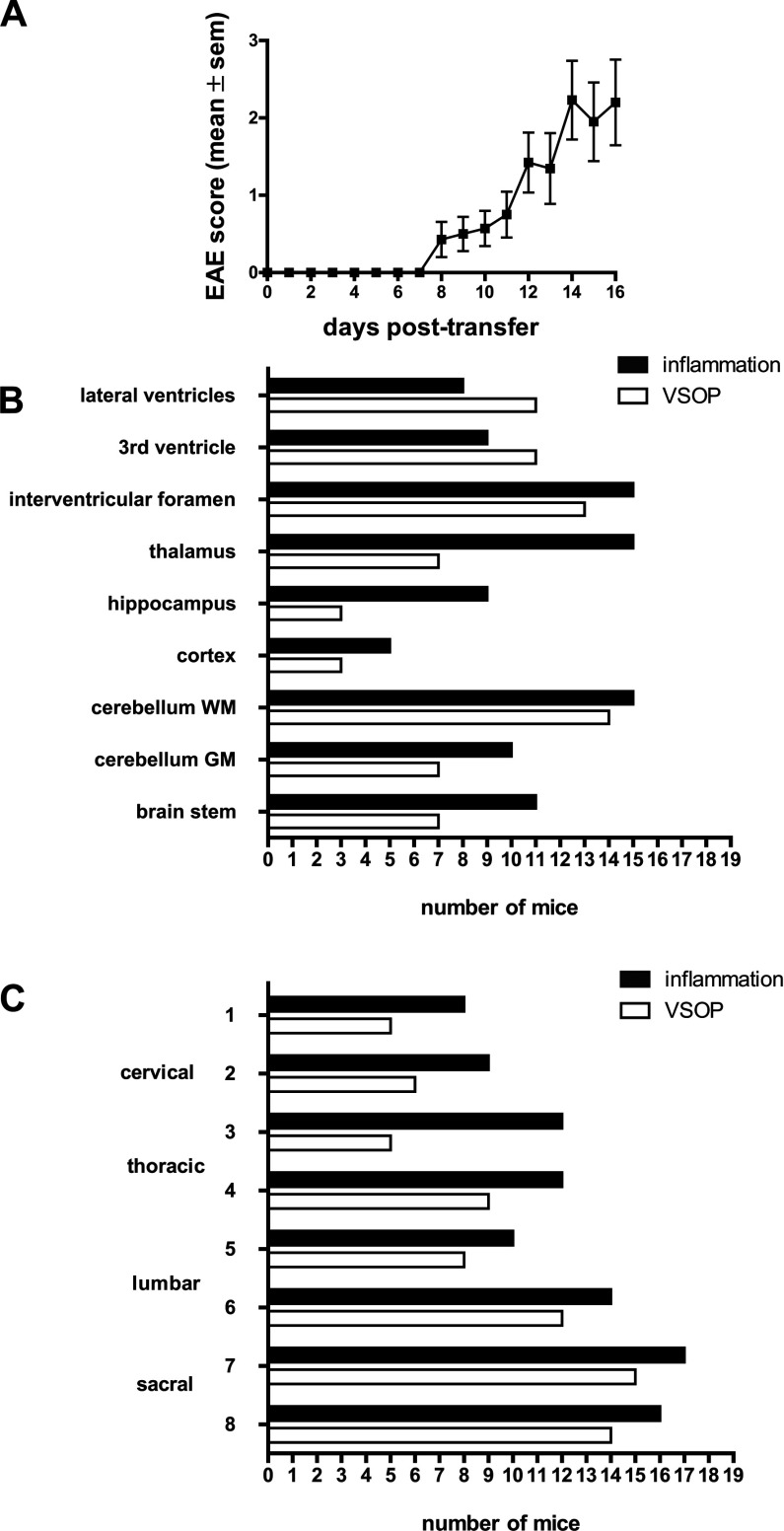
Histological detection of inflammation and VSOP at peak disease (**A**) Plot of EAE clinical score in SJL mice after adoptive transfer of PLP1_39-151_ reactive T-cells. (**B**) Number of mice showing inflammatory lesions (pathological accumulations of immune cells) and VSOP in various brain regions. (**C**) Distribution of inflammatory lesions and VSOP in transverse spinal cord segments. *n*=19.

To better understand the involvement of choroid plexus and spinal cord meninges in EAE development, we then examined the distribution of VSOP at peak EAE, particularly in the choroid plexus and in the inflamed spinal cord. EAE was induced in SJL wild-type recipient mice by adoptive transfer of encephalitogenic T-cells from PLP_139-151_-immunized donor mice. The typical EAE disease course expected with this model was observed, as shown in Figure 3(A). Disease onset occurred between 8 and 12 days after adoptive transfer of T-cells, and peaked at days 14–16 post transfer. The distribution of VSOP and inflammatory lesions (defined as a pathological accumulation of immune cell infiltrates as shown by H&E staining) is shown in Figures 3(B) and 3(C) for brain and spinal cord, respectively. Inflammatory lesions were observed in the thalamus and hippocampus, and less frequently in the cortex. Lesions were especially prominent in the cerebellum white matter tracts, and also present in cerebellar grey matter and in the brainstem. VSOP could be detected in lesions in all of these regions. Of particular note was the prevalence of VSOP observed in the choroid plexus, both in the ventricles and extending into the interventricular foramen (Figure 3B). We observed VSOP in both mild and severe lesions. However, we also saw both mild and severe lesions where VSOP was absent. Thus, the presence of VSOP does not appear to be correlated with the magnitude of the individual lesions.

**Figure 4:**
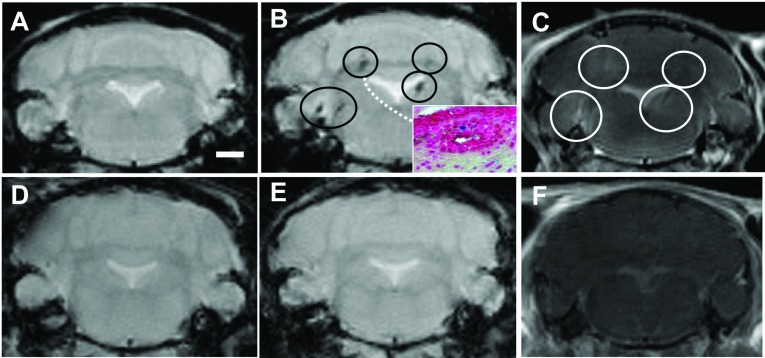
VSOP location in brain correlates with T2*-enhancing lesions Representative T2*-weighted images prior to contrast agent administration are shown for EAE (**A**) and healthy control (**D**) brains. Hypointense lesions (circles) are seen in the T2*-weighted image 24 h post-VSOP administration in the EAE brain (**B**) but not in the control (**E**). Diffuse hyperintense Gd-DTPA-enhancing lesions are seen in representative T1-weighted images in the EAE brain (**C**) but not in the healthy control (**F**). MRI-detected VSOP-enhancing lesions were verified by histology, showing the presence of VSOP (blue) in inflamed lesions (inset in **B**). Scale bar=1 mm.

The correspondence between VSOP enhancement on MRI and histological detection was verified in 16 animals; these mice first underwent T1- and T2*-weighted scans prior to the application of contrast agents. After this, the mice were administered intravenous Gd-DTPA, and scanned immediately again by T1-weighted MRI. Subsequently, the mice were administered intravenous VSOP, then scanned for T2*-weighted MRI 24 h later. The 24 h time point was based on our previous studies which showed that by 24 h post-VSOP injection, the blood–pool contrast had entirely subsided, leaving the pathological VSOP-enhancing lesions clearly discernible (Tysiak et al., 2009; Wuerfel et al., 2007). A representative MRI example of a mouse with severe disease (bilateral hind limb paralysis) is shown in Figure 4(A) prior to the application of VSOP. Hypointense lesions are visible in the cerebellum and brainstem in the post-VSOP T2*-weighted image shown in Figure 4(B), which are not discernable in the pre-contrast scan in Figure 4(A). These lesions coincided with Gd-DTPA-enhancing hyperintense lesions (Figure 4C). After the post-VSOP MRI scanning, the mice were killed and processed for histology. The inset in Figure 4(B) shows the histological detection of VSOP in an inflammatory lesion in the cerebellar white matter, corresponding to a lesion shown in the same mouse by the MRI scans. Figure 4(D) shows a representative example of a healthy control mouse pre-contrast. No hypointense or hyperintense lesions were seen in this control mouse after application of VSOP (Figure 4E) or Gd-DTPA (Figure 4F).

**Figure 5:**
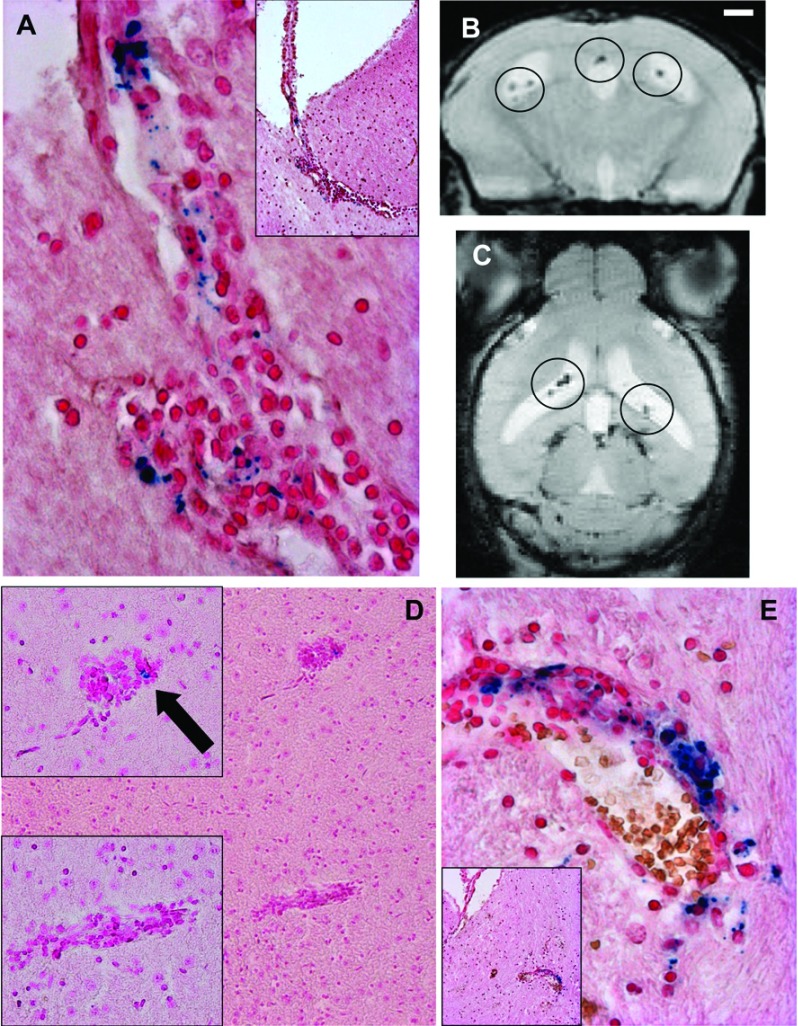
VSOP observed in both severe and mild inflammatory lesions At peak disease VSOP was detected by histology in inflamed choroid plexus extending from the lateral ventricle into the interventricular foramen (**A**), and could also be seen as hypointensities on coronal (**B**) and axial (**C**) T2*-weighted images. Representative examples of mild inflammatory lesions that were both VSOP-positive (arrow) and VSOP-negative were seen in brain parenchyma (**D**). A representative example of a more severe inflammatory lesion showing the presence of VSOP as well as erythrocytes (brown) is shown in (**E**). Original magnification: (**A**, **E**) ×200; (**D**) ×100. Scale bar (**B**)=1 mm.

Figure 5(A) shows a representative staining of VSOP in inflamed choroid plexus, extending from the lateral ventricle into the interventricular foramen. Note that the morphology of the tissue structure is highly disrupted by the infiltrating immune cells. VSOP could also be detected by MRI in choroid plexus as hypointensities within the ventricles, as shown in Figures 5(B) and 5(C) (coronal and axial views, respectively). At peak of disease, numerous lesions were observed at various stages of development. Figure 5(E) shows a representative example of relatively mild lesions in the brain parenchyma (thalamus). VSOP was observed in some of these lesions (arrow in Figure 5E), although VSOP-negative lesions were also detected. An example of a more severe lesion with prominent VSOP is shown in Figure 5(F). In this example, erythrocytes can be seen in the tissue (brown colour). The observation of both VSOP and erythrocytes in the lesion, but with different colours, underscores the specificity of the Prussian Blue staining method for the iron of the VSOP.

### Detection of VSOP in lesions in inflamed spinal cord

In the present study, we addressed the distribution of VSOP in spinal cord of mice at peak EAE by histology. As depicted in Figure 3(C), inflammatory pathology and VSOP could be observed throughout the spinal cord. The VSOP was especially prominent in the meninges, the anterior median fissure and the posterior median sulcus, and in white matter lesions. Grey matter lesions were only occasionally observed, and no VSOP was detected in the central canal. At peak disease, detection of VSOP in spinal cord always corresponded with inflammatory pathology but not vice versa, i.e. inflammatory lesions could also be seen without accompanying VSOP.

**Figure 6:**
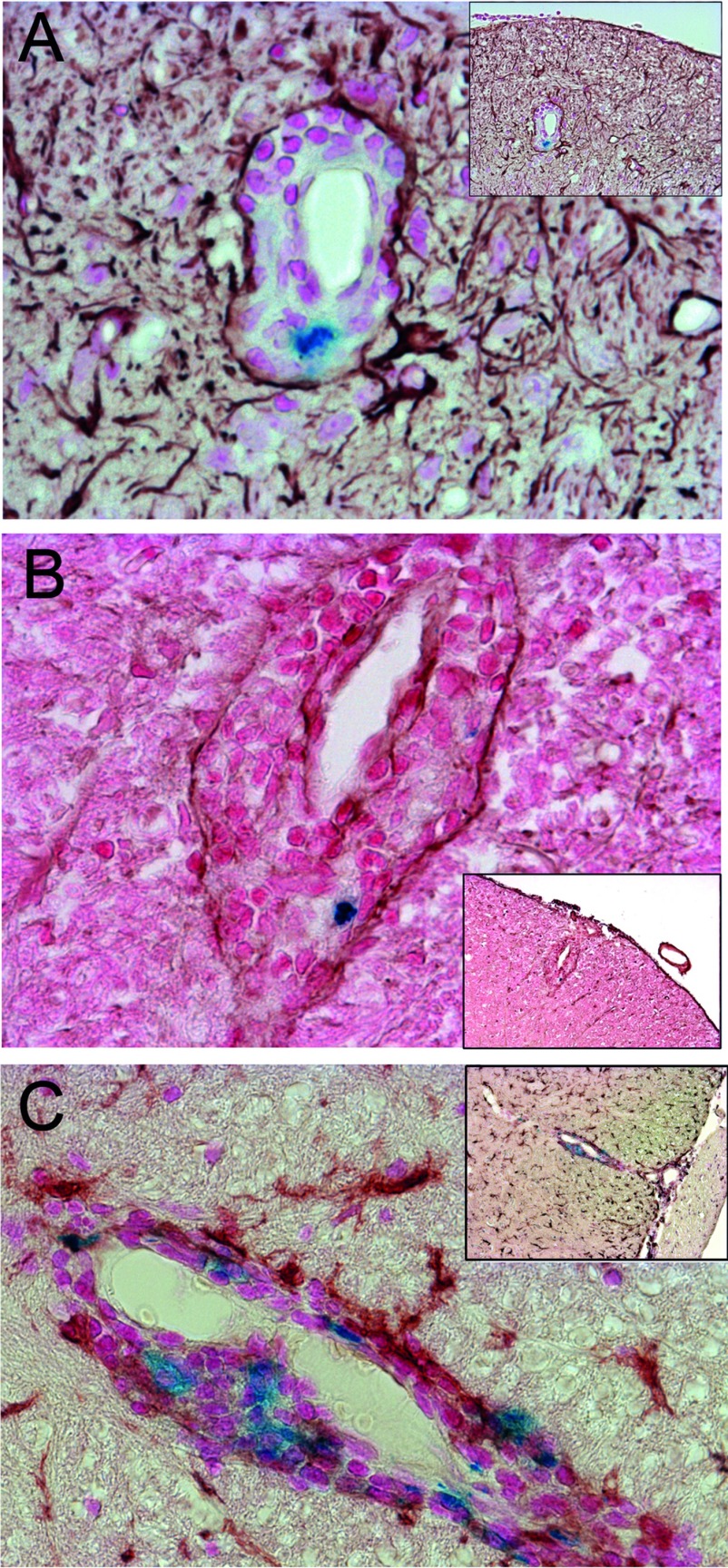
VSOP observed in perivascular-restricted spinal cord lesions with intact BBB (**A**) GFAP immunostaining (brown) shows astrocyte end foot processes surrounding a lesion with immune infiltrates (stained with Nuclear Fast Red) and including VSOP (blue). (**B**) Immunostaining for laminin (brown) shows vascular endothelium and glia limitans of a perivascular lesion, along with infiltrating cells and VSOP (blue). (**C**) Immunostaining for iba-1 (brown) shows the presence of microglia in CNS parenchyma, and activated microglia/macrophages associated with VSOP (blue) located in the perivascular lesion. Original magnification: (**A**–**C**) ×200.

To further dissect the VSOP distribution in sick animals, we used immunohistochemistry combined with Prussian Blue staining to detect VSOP *in situ* in relation to other cellular and non-cellular elements in inflammatory lesions. In Figure 6(A), we show an exemplary inflammatory lesion in which the infiltrating immune cells are restricted to the perivascular space. Immunostaining with GFAP clearly shows the astrocyte end processes delineating the glia limitans surrounding the lesion. In this example, the infiltrating cells and the VSOP are confined to the perivascular space, and have not entered the parenchyma of the CNS proper. Similarly, in Figure 6(B), immunostaining with a pan-laminin antibody delineates the vascular endothelial and the parenchymal basement membranes; in this lesion, the infiltrating cells and the VSOP are also confined to the perivascular space.

To investigate the association of VSOP with phagocytes we did immunostaining for iba-1. In Figure 6(C), iba-1 immunostaining shows microglia with the typical ramified morphology in the spinal cord parenchyma, and iba-1-positive cells co-localized with VSOP in the inflammatory lesion. In addition to perivascular lesions, disseminated lesions with VSOP and immune cell infiltration into the parenchyma were seen in both spinal cord and brain at peak disease.

### Association of VSOP with endothelium in inflammatory lesions

**Figure 7:**
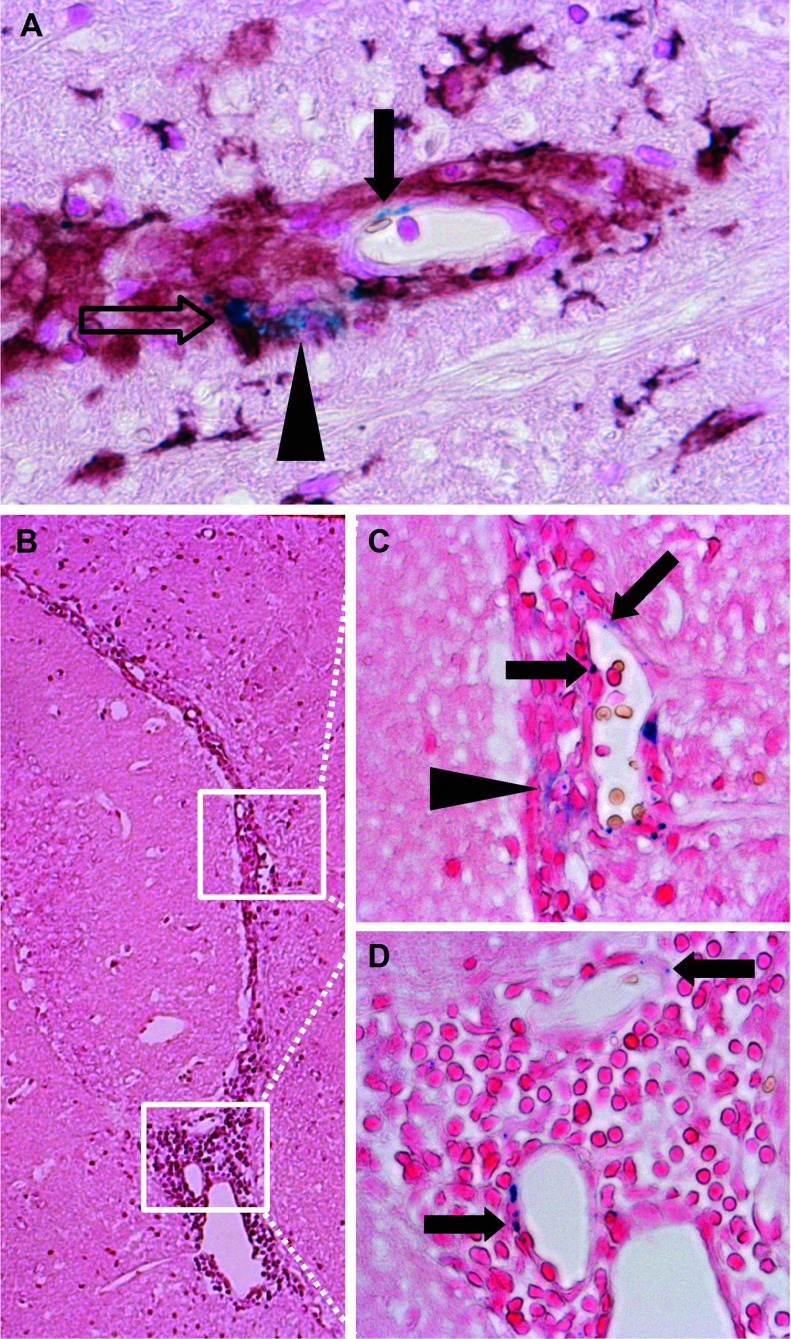
VSOP present in multiple forms in CNS lesions at peak disease VSOP could be seen as discrete puncta that appear to be present in elongated endothelial structures (solid arrow in **A**). VSOP was also seen in the same lesion co-localized with an iba-1-positive cell (open arrow in **A**), and as a diffuse accumulation (arrowhead in **A**). Multiple forms of VSOP in inflamed choroid plexus in the interventricular foramen (**B**). VSOP as discrete puncta structures (solid arrows in **C** and **D**), and as a diffuse accumulation (arrowhead in **C**). Original magnification: (**A**, **C**, **D**) ×200; (**B**) ×100.

Apart from the detection of VSOP in perivascular lesions, in the present study, we observed for the first time VSOP in spinal cord inflammatory lesions as discrete puncta present in elongated structures, which appear to be vascular endothelium (solid arrow in Figure 7A). Endothelial VSOP could be seen in close proximity to VSOP co-localized with iba-1-positive cells in the same lesion (open arrow in Figure 7A). VSOP was also detected in lesions as a diffuse accumulation that did not appear to be cell-associated (arrowhead in Figure 7A). Diffuse, non-cell-associated VSOP was also seen in the brain, as shown in the inflamed choroid plexus in the interventricular foramen in Figure 7(B). The higher magnification views in Figures 7(C) and 7(D) show discrete punctate VSOP located within elongated structures which appear to be the vascular endothelium of the choroid plexus. Additional diffuse VSOP was seen in the choroid plexus (Figure 7C, arrowhead), although it is not possible to determine whether this non-cell-associated VSOP is located within the stroma, given the substantial disruption of the tissue architecture resulting from the accumulation of immune cells.

## DISCUSSION

In the present study, we demonstrated that VSOP accumulated in the choroid plexus and spinal cord meninges of asymptomatic mice after adoptive transfer of encephalitogenic T-cells, prior to the onset of clinical signs and in the absence of overt inflammation. VSOP appears to reveal early EAE-related inflammatory alterations at the choroid plexus explicitly. Since VSOP was completely absent from the choroid plexus of non-immunized healthy animals without T-cell transfer, our data point to choroid plexus involvement not only in physiological immune surveillance and during established CNS inflammation (Engelhardt and Ransohoff, 2005; Ransohoff et al., 2003; Reboldi et al., 2009; Vercellino et al., 2008) but also as a site for early events in the pathogenesis of CNS autoimmunity.

VSOP detected in the choroid plexus prior to EAE onset appeared as discrete foci located in the choroid plexus stroma. In some cases the morphology of the plexus tissue remained intact, with no obvious accumulation of infiltrating immune cells in this early disease phase (Figures 2A and 2B), while in other cases, mild inflammation was seen (Figure 2C). In contrast, the morphology of the choroid plexus at peak disease was highly disrupted (Figures 4D and 7B–7D). It is not yet clear which structures in the choroid plexus of the asymptomatic mice are associated with VSOP. The choroid plexus endothelium is fenestrated, which in principle could permit the diffusion of free VSOP from the circulation into the stroma. However, no VSOP was detected in the choroid plexus (or indeed anywhere else in the CNS) of unmanipulated SJL mice in which the same dose of VSOP was administered before processing for histology. Thus, VSOP accumulation in the choroid plexus stroma cannot be explained by mere diffusion mechanisms. On the other hand, VSOP could be contained within phagocytes that had entered from the periphery at this early EAE phase. However, we did not observe VSOP co-localized with iba-1-positive structures in the choroid plexus in asymptomatic animals (data not shown). Thus, mechanisms other than physiological diffusion processes or early migration of inflammatory phagocytes from the periphery may explain VSOP accumulation in choroid plexus (Wolburg and Paulus, 2010). It may be that as a consequence of the systemic inflammation induced by adoptive transfer, early circulating inflammatory factors (Mitchell et al., 2009) may alter resident myeloid cells in the choroid plexus stroma (Nataf et al., 2006), leading to the observed accumulation of VSOP in asymptomatic mice. Future studies using additional immunohistochemical markers should address these possibilities in detail.

Similar to the choroid plexus, VSOP was also observed in the spinal cord meninges in pre-onset mice in the absence of overt inflammation but not in naive mice given VSOP, suggesting that this observation reflected early events in the disease process. It is proposed that immune cells gain entry to the CNS via the choroid plexus, circulate through the CSF (cerebrospinal fluid) compartment, including the subarachnoid space, as part of the process of immune surveillance of the CNS, and can encounter antigen and be re-activated in the meninges during the earliest stages of neuroinflammation (Brown and Sawchenko, 2007; Reboldi et al., 2009). Indeed, it was reported that CD4+ Th1 and Th17 cells accumulate and become re-activated in the subarachnoid space prior to entry into spinal cord parenchyma, and prior to clinical onset of EAE (Kivisakk et al., 2009). The accumulation of VSOP in the choroid plexus and spinal cord meninges at the first stages in the disease process confirms that these structures are altered very early during neuroinflammation.

While in asymptomatic animals VSOP highlighted the involvement of choroid plexus and meninges in early inflammatory events, VSOP in animals at peak disease showed a broad and heterogeneous distribution. At peak disease, VSOP was seen in the choroid plexus in 15 out of 19 animals (79%) and could be clearly visualized by MRI, in contrast with the choroid plexus VSOP in asymptomatic animals which was not detectable by MRI. Most likely this is a question of sensitivity, and work is in progress to determine the limit of detection of VSOP by MRI *in vivo*. One possibility to address this would be to use MRI scanners with greater B0 field strength. We recently reported detection of EAE lesions using a 9.4 T MRI scanner with a cryogenically cooled coil (Waiczies et al., 2012), and it would be interesting to apply this higher B0 field strength to studies with VSOP, to attempt to approach the sensitivity of histological detection of VSOP *in vivo*. As presented in Figures 5(A) and 7(B)–(D), the inflamed choroid plexus has a highly disrupted morphology, consistent with previous reports in severe EAE (Engelhardt et al., 2001; Murugesan et al., 2012).

At peak disease, we also show histological evidence for the accumulation of VSOP in perivascular inflammatory lesions with intact glia limitans. This type of perivascular accumulation of immune cells may represent a relatively early and potentially reversible stage in the development of a CNS lesion, prior to entry of the infiltrating cells into the brain parenchyma, where they may cause tissue damage. In principle, early detection of such lesions may be instrumental in the development of novel therapeutic strategies that target these distinct mechanisms and may offer the opportunity for earlier clinical intervention (Wuerfel et al., 2008).

Moreover, in diseased mice, we observed within the same perivascular lesion VSOP as discrete focal accumulations that appear to be co-localized with iba-1-positive cells, as well as diffuse VSOP accumulations which did not appear to be cell-associated. It has been well established that iron oxide particles are efficiently taken up by phagocytic cells (Rausch et al., 2003, 2004; Weinstein et al., 2010). As described for large iron oxide particles (Weinstein et al., 2010), smaller VSOP appear also to be readily taken up by phagocytic cells such as macrophages (Stroh et al., 2006; Fleige et al., 2002), despite their small diameter (7 nm) (Wuerfel et al., 2007). Uptake of VSOP by monocytes and macrophages has been confirmed using electron microscopy (Ludwig et al., 2013). Thus, VSOP has the potential to enter CNS tissue both associated with phagocytes and in a non-cell-associated form, which in principle could highlight distinct aspects of the pathological process. Furthermore, we observed for the first time VSOP as discrete puncta that seem to be present in endothelial cells. This apparent endothelial incorporation was seen both in lesions in the brain and spinal cord parenchyma, as well as in the choroid plexus, at peak disease. Activation of vascular endothelium in response to inflammatory stimuli is associated with up-regulation of numerous adhesion molecules, as well as extracellular matrix elements such as hyaluronic acid (Nandi et al., 2000), which may facilitate electrostatic interactions with VSOP. Indeed, it was recently demonstrated that VSOP can bind strongly to cell membranes via interactions with negatively-charged GAGs (glycosaminoglycans) (Ludwig et al., 2013). Alteration of GAG expression during inflammation, both in the vascular endothelium and in the choroid plexus, may underlie interactions of VSOP in these structure, though the specific molecular mechanisms of such putative interactions remain to be investigated in future studies, including the use of *in vitro* BBB and BCSFB (blood–CSF barrier) model systems. Additionally, work is in progress to employ a SQUID (superconducting quantum interference device) to precisely measure iron content by magnetorelaxometry in tissues *ex vivo*, after application of VSOP (Eberbeck et al., 2008; Richter et al., 2010). The use of SQUID will enable a quantitative determination of the distribution of VSOP to various tissue compartments, which can be compared between healthy controls and EAE mice at different time points during disease.

One caveat of the present study is the lack of an established fluorescent method for histological detection of iron, which precludes the use of confocal microscopy to definitively establish cellular co-localization and internalization of VSOP. Work is in progress to circumvent this limitation by the development of next generation VSOP that incorporate europium atoms directly into the iron oxide core. This renders the particles detectable by both MRI and fluorescence (Groman et al., 2007), and will permit future studies to make more conclusive statements regarding cellular and extracellular localization *in vivo*. In particular, immunostaining with specific markers for endothelial cells and phagocytic cells combined with fluorescent VSOP will facilitate corroboration of our initial findings for VSOP in the endothelium and choroid plexus, respectively.

In summary, in the present study, using VSOP, we demonstrated that apart from its involvement in immune surveillance, the choroid plexus is involved in early inflammatory processes in autoimmune inflammation. It seems to undergo alterations occurring very early in the process of inflammation that enables the specific binding of VSOP to stromal structures of the choroid plexus. On the other hand, in established EAE, we show accumulation of VSOP both in CNS perivascular lesions and associated with endothelial cells, two processes which may represent an early stage in the pathological cascade in EAE. Thus, VSOP may emerge as a useful complement to conventional MRI approaches to monitor early cellular changes in the context of inflammation. VSOP has already been applied in human clinical studies in the context of MR angiography (Taupitz et al., 2004; Wagner et al., 2011). It is therefore essential that we expand our understanding of how these particles interact with cellular and non-cellular elements *in vivo*. Importantly, the use of VSOP will help improve our understanding of the pathomechanisms in multiple sclerosis and other inflammatory disorders of the CNS.
